# E-cadherin downregulation sensitizes *PTEN*-mutant tumors to PI3Kβ silencing

**DOI:** 10.18632/oncotarget.13414

**Published:** 2016-11-16

**Authors:** África Millán-Uclés, Susana Zuluaga, Miriam Marqués, Jesus Vallejo-Díaz, Lorena Sanz, Ariel E Cariaga-Martínez, Francisco X Real, Ana C. Carrera

**Affiliations:** ^1^ Department of Immunology and Oncology, Centro Nacional de Biotecnología/CSIC, Universidad Autónoma de Madrid, Cantoblanco, Madrid, Spain; ^2^ Centro Nacional de Investigaciones Oncológicas, Melchor Fernández Almagro 3, Madrid, Spain; ^3^ Departament de Ciències Experimentals i de la Salut, Universitat Pompeu Fabra, Barcelona, Spain

**Keywords:** PI3Kbeta, PTEN, urothelial carcinoma, bladder cancer, siRNA

## Abstract

Alterations in phosphatidylinositol 3-kinase (PI3K) and in PTEN (phosphatase and tensin homolog), the negative regulator of the PI3K pathway, are found in nearly half of human tumors. As PI3Kβ, the main isoform activated in *PTEN-*mutant tumors, has kinase-dependent and -independent activities, we compared the effects of depleting vs. drug-inhibiting PI3Kβ kinase activity in a collection of diverse tumor types and in a set of bladder carcinoma cell lines grown as xenografts in mice. PI3Kβ depletion (by intratumor injection of *PIK3CB* siRNA) induced apoptosis and triggered regression of *PTEN*-mutant tumors more efficiently than PI3Kβ inhibition. A small proportion of these tumors was resistant to PI3Kβ downregulation; we analyzed what determined resistance in these cases. Using add-back experiments, we show that both *PTEN* mutation and low E-cadherin expression are necessary for PI3Kβ dependence. In bladder carcinoma, loss of E-cadherin expression coincides with N-cadherin upregulation. We found that PI3Kβ associated with N-cadherin and that *PIK3CB* depletion selectively disrupted N-cadherin cell adhesions in *PTEN*-mutant bladder carcinoma. These results support the use of *PIK3CB* interfering RNA as a therapeutic approach for high-risk bladder cancers that show E-cadherin loss and express mutant *PTEN*.

## Introduction

Enhanced activity of the class I_A_ phosphoinositide 3-kinase (PI3K) pathway is frequent in cancer [1-6]. PI3K are lipid kinases encoded by *PIK3* genes, of which *PIK3CA* and* CB* are ubiquitous [1-6]. After activation by growth factors, PI3K generate phosphoinositide (PI)(3,4,5)P_3_ at the plasma membrane; this stimulates downstream targets such as PKB and mTOR, which trigger cell survival, division, and migration. The phosphatase PTEN reduces PI(3,4,5)P_3_ levels [6]. *PIK3CA* mutations appear at early stages in cancer progression*,* whereas* PTEN* inactivation is a later event [3-10]. The role of PI3Kβ in cancer is less understood; *PIK3CB* mutations in cancer appear at lower frequency than those of *PIK3CA* [5, 11], but enhanced PI3Kβ expression is sufficient to induce transformation [12]. PI3Kβ levels are increased in colon, ovarian, endometrial, breast and bladder carcinoma [3, 5, 9, 10].

In the mouse, expression of active PI3Kβ leads to intraepithelial prostate neoplasia and *Pik3cb* deletion inhibits prostate cancer in *Pten*^+/-^ mice [13, 14]. These observations led to studies in xenograft models, which showed that PI3Kβ inhibitors decelerate tumor growth in *PTEN*-mutant tumors [15-17]. The correlation between *PTEN* loss of function and PI3Kβ inhibitor-mediated cytostatic effect is not universal, and the mechanism underlying *PTEN* and PI3Kβ cooperation is unclear [5, 18-20]. PI3K is considered a key target for cancer therapy, but only enzymatic inhibitors have been tested clinically [21-23]. Some of these studies are promising, but others have failed due to blockade of feedback mechanisms or acquisition of resistance [5, 21-23]. Since PI3Kβ generates PI(3,4,5)P_3_ but also has kinase-independent functions [24-26], we tested the effect of depleting PI3Kβ using interfering RNA (siRNA) on a collection of diverse tumor types and in a set of urothelial bladder carcinoma (UBC) lines.

UBC is the fifth most frequent tumor in developed countries; although the majority of these tumors (75%) are non muscle-invasive (NMI) and treatable by transurethral resection, many recur (70%) and up to 10-15% progress to muscle-invasive (MI) metastatic carcinoma, with poor prognosis [1, 2, 5. The molecular mechanisms that underlie UBC invasiveness must thus be identified, as well as new therapeutic strategies for MI-UBC.

We tested the effect of depleting PI3Kβ in these tumors, since UBC tumors show enhanced PI3Kβ expression, loss of *PTEN* function at advanced stages, and accessibility for local administration of siRNA [4, 7, 10]. We show that PI3Kβ depletion is cytotoxic for most tumors with mutant* PTEN*. Treatment of *PTEN*-mutant tumor xenografts by intratumor (i.t.) injection of *PIK3CB* siRNA (si*PIK3CB*) or by expression of doxycycline (doxy)-inducible *PIK3CB* shRNA was markedly more potent than PI3Kβ inhibition. Some *PTEN*-mutant tumor cells were resistant to si*PIK3CB*; we took advantage of this phenotype to study what determines resistance. We show that *PTEN* inactivation and E-cadherin (E-cad) loss are necessary for sensitivity. These observations support development of *PIK3CB*-interfering approaches for treatment of advanced UBC with E-cad loss and *PTEN* mutations.

## Results

### *PTEN* mutation is not sufficient for tumor PI3Kβ dependence

PI3Kβ enzymatic inhibition retards the growth of most *PTEN*-mutant tumor xenografts, but does not trigger cell death [16, 18-20]. Since PI3Kβ has enzymatic and non-enzymatic activities [24-26], we evaluated the effect of depleting *PIK3CB* in xenografts. We optimized PI3Kβ depletion using siRNA in *PTEN-*mutant prostate cancer PC3 cells. Transfection of 0.5x10^6^ cells with 6 to 12 pmol of siRNA1 or siRNA2 was sufficient for efficient silencing and for reduction of cell viability (Supplementary Figure S1a). We also grew PC3 cell-derived xenografts in immunodeficient mice; when tumors reached ~75 mm^3^, mice were treated with siRNA. As systemic siRNA administration by intraperitoneal injection (i.p.) did not deplete PI3Kβ efficiently in tumors (not shown), we optimized administration by direct injection into the tumor center of *PIK3CB* siRNA1 (si*PIK3CB*1) (12.5, 50, 120 pmol/mm^3^ tumor) on days 1, 3 and 5, and effects were tested on day 7. The 50pmol/mm^3^ dose of si*PIK3CB*1optimally reduced PI3Kβ levels in PC3 xenografts and the PI3Kβ -dependent [25] PCNA binding to chromatin (Supplementary Figure S1b).

To study the effect of PI3K depletion on PC3 tumor growth, we divided a set of mice bearing ~75 mm^3^ tumors into random groups, which we treated with PBS, liposomes, or liposomes plus control or si*PIK3CB*1 (50pmol/mm^3^). Treatment was administered three times a week for two weeks. Repeated injection with liposomes decelerated tumor growth; nonetheless, si*PIK3CB* delivery triggered tumor regression (Supplementary Figure S1c). siRNA delivery at the end of treatment might have been suboptimal due to the small tumor size; optimal *PIK3CB* depletion might have yielded additional effects. Using representative PC3 tumor extracts, we showed that PI3Kβ knockdown reduced pPKB levels and PCNA binding to chromatin (Supplementary Figure S1d). Histopathology analysis of representative samples showed that whereas necrotic areas in vehicle and control siRNA-treated tumors did not exceed 10% of the tumor, the cell death index in si*PIK3CB1*-treated tumors reached 90-100% of cells in 60% of samples; all liposome-treated samples showed an inflammatory neutrophil infiltration, which might explain the tumor growth deceleration in the presence of vehicle (Supplementary Figure S1e). TUNEL staining of tumor sections showed that those treated with control siRNA had few positive cells, and that si*PIK3CB1* treatment significantly increased TUNEL^+^ cells (Supplementary Figure S1f).

**Figure 1:**
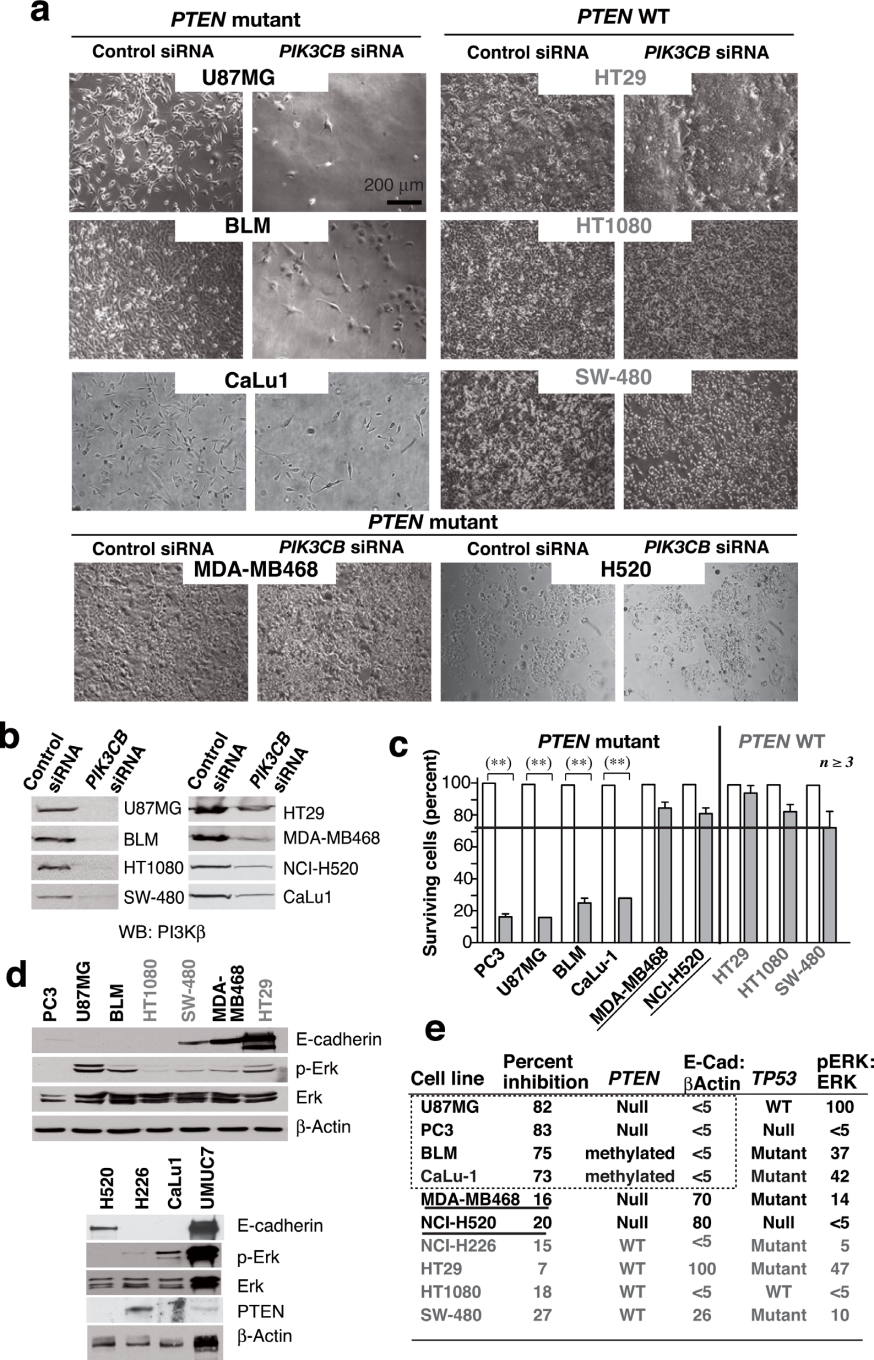
***PTEN*** mutation is necessary but not sufficient for PI3Kβ dependence of cancer cell lines. **a**. We transfected various WT or *PTEN* mutant cancer cell lines (0.5x10^6^ cells) with 12 or 20 pmol si*PIK3CB* (depending on cell line) on days 1 and 3 and examined them at day 5. Representative images for 8 cell lines tested. **b**. Western blot (WB) analysis of extracts from tumor cells as in **a**.. **c**. Viability of cells treated with si*PIK3CB* as in **a**. and compared to controls (100%). ** *P* < 0.01, Student's *t*-test (*n* ≥ 3). **d**. Extracts of various tumor cells were tested in WB (indicated). **e**. Summary of tumor cell responses to PI3Kβ silencing (as a percent of cell survival inhibition), the *TP53* and *PTEN* mutation status, and the ratio of pErk:Erk or E-cad:β-actin signals in **d**. expressed as a percentage of maximum levels (100%). Cell lines with mutant *PTEN* and high E-cad levels are underlined.

We extended the analysis to a collection of cell lines representative of distinct tumor types that expressed wild-type (WT) *PTEN* (HT-29, SW480, NCI-H226, HT1080), mutant *PTEN* (U87MG, MDA-MB468, BLM, H520), or showed *PTEN* promoter methylation (CaLu-1) [27]. We performed two transfections (days 1 and 3) using si*PIK3CB*1 (0.2 nmol/0.5x10^6^ cells) and tested the consequences on day 5. *PIK3CB* silencing did not affect WT *PTEN* lines but reduced viability of the cells with* PTEN* loss of function, with the exception of MDA-MB468 and H520 cells, which were resistant (Figure 1a-1c). This shows that *PTEN* inactivation is necessary but insufficient for PI3Kβ dependence.

To define which pathways could synergize with *PTEN* inactivation to render the cells PI3Kβ-dependent, we examined features frequently altered in cancer, including E-cad levels, *TP53* mutation and ERK activation [28, 29]. All cells were pre-checked for *TP53* mutations ( cansar.icr.uk/cansar/cell lines); we used Western blot (WB) to evaluate pERK and E-cad levels (Figure 1d). The si*PIK3CB*-responsive cells shared *PTEN* mutation and low E-cad levels but presented variable pERK and *TP53* status; accordingly, the two *PTEN* mutant resistant cells (MDA-MB468 and H520) expressed high E-cad levels (Figure 1d, 1e).

### *PTEN* mutation is not sufficient for PI3Kβ dependence in bladder cancer

**Figure 2:**
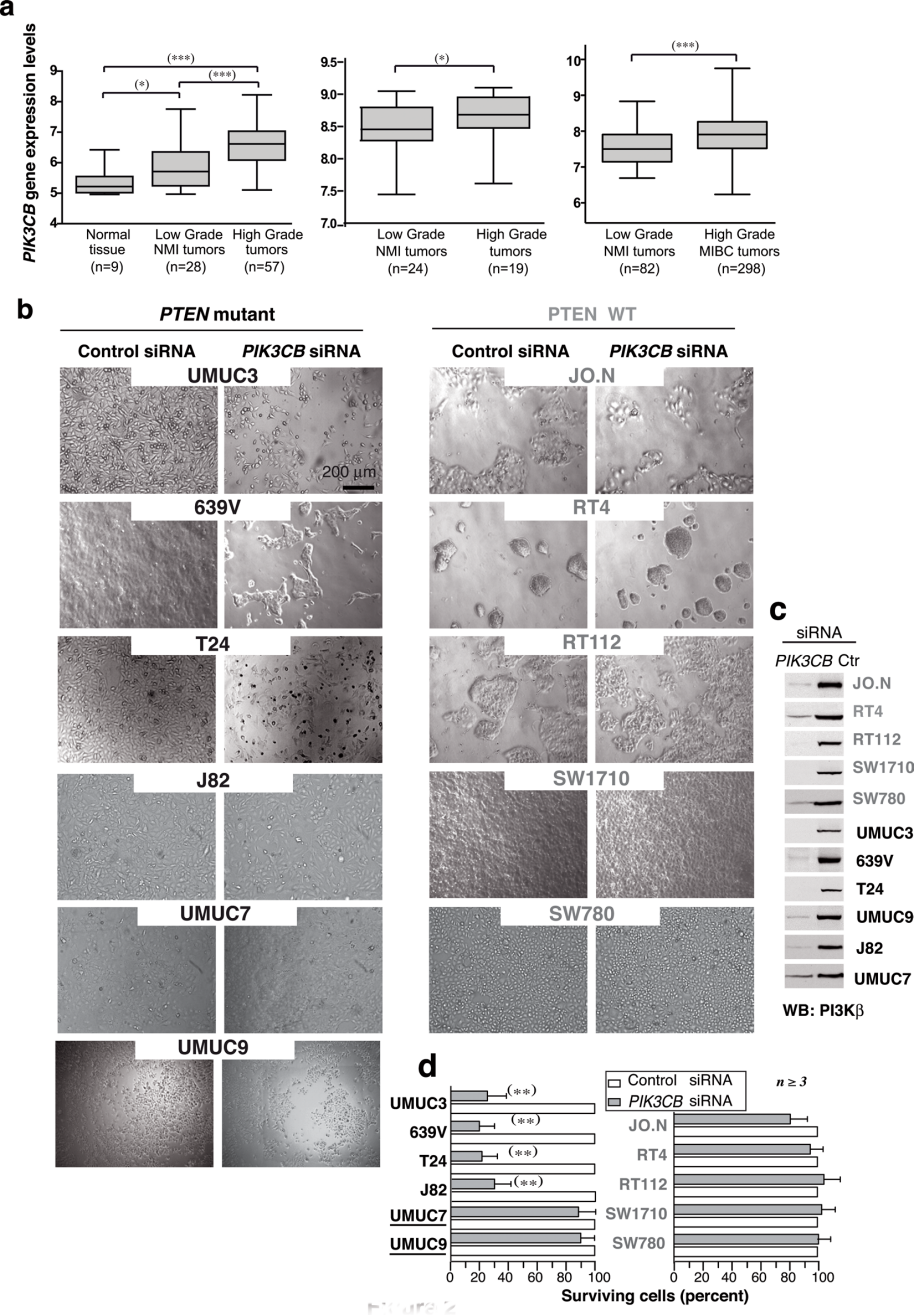
Advanced clinical UBC express high ***PIK3CB*** levels; ***PIK3CB*** depletion reduces cell survival in UBC lines. **a**. Box plots show *PIK3CB* gene expression (mean ± SEM) in clinical UBC. Gene expression data are expressed in log2 Robust Multichip Average (RMA) intensity units. Corresponding GEO accession numbers are GSE5287 (left panel) and GSM1838824 (center). The third dataset (right) is available from ArrayExpress (see Methods). MI, muscle-invasive; NMI, non-muscle invasive. For statistical analysis, we used the Kruskal-Wallis test with two-tailed Mann-Whitney post hoc test for paired group comparison; the one-tailed Mann-Whitney test was applied for group comparison. *** *P* ≤0.0001, ** *P* < 0.01, * *P* < 0.05. (b, c) Bladder carcinoma cell lines (0.5 x 10^6^ cells) were transfected on days 1 and 3 with 0.2 nmol control or si*PIK3CB* and were analyzed on day 5. Images show representative fields **b**. and WB analysis of tumor extracts **c**.. **d**. Percentage of surviving cells at experiment termination in si*PIK3CB*- *vs*. control siRNA-transfected cells (100%). ** *P* < 0.01 Student's *t*-test.

Previous studies using a few cell lines suggest that PI3Kβ expression increases in UBC [9]; we examined a large UBC collection from reported DNA microarrays [30-32]. Samples were grouped as “normal tissue”, “low-grade NMI” (G1-2, Ta-T1), and “high-grade tumors” including NMI (G3, Ta-T2) and MI tumors (T3-T4). “Low-grade tumors” had higher *PIK3CB* mRNA levels than normal bladder tissue, and “high-grade tumors” had the highest levels (Figure 2a). Data were confirmed in two independent datasets; *PIK3CB* gene expression levels were higher in “high-grade” than in “low-grade” tumors [31, 32] (Figure 2a). These results show that increased *PIK3CB* expression is frequent at advanced stages UBC.

We next studied 11 well-characterized UBC lines [33]. We optimized si*PIK3CB* treatment in 639V UBC cells (Supplementary Figure S2a-S2f)*;* transfection (0.5x10^6^ cells) on day 1 and 3 with 0.2 nmol si*PIK3CB1* efficiently depleted PI3Kβ by day 5 and reduced cell viability (Figure 2b-2d), expression of *PIK3CB* shRNA had a similar effect (Supplementary Figure S2a-S2f). WT *PTEN* lines were resistant to *PIK3CB* silencing; of the six *PTEN*-mutant or -low-expression lines, four were sensitive, but UMUC-9 and -7 cells were resistant to PI3Kβ depletion (Figure 2b-2d) confirming that *PTEN* loss is insufficient for PI3Kβ dependency.

**Figure 3:**
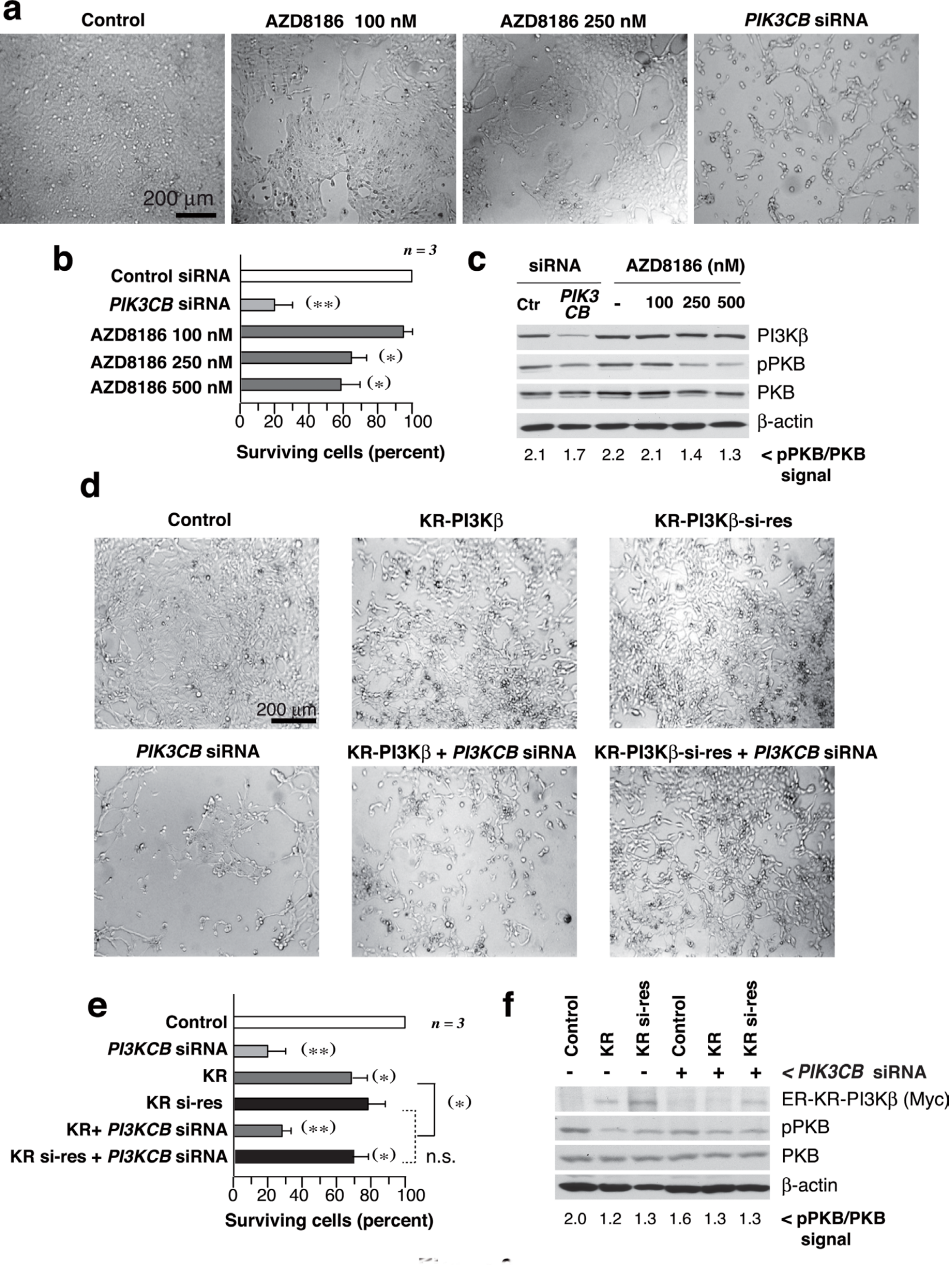
PI3Kβ regulates UBC cell survival in a kinase-independent manner **a**.-**c**. 639V cells were cultured with AZD8186 (dose indicated) or were transfected with si*PIK3CB* at days 1 and 3, and examined at day 5. Images show representative fields **a**., percentage of surviving cells at experiment termination compared to control cells (100%) **b**., and WB examination of cell extracts **c**.; pPKB signal normalized to PKB is indicated at the bottom **c**.. **d**., **e**. 639V cells were transfected with control or si*PIK3CB* alone or in combination with ER-myc-K805R-p110β (KR-PI3Kβ) or the si*PIK3CB*-resistant mutant (KR-PI3Kβ-si-res) (72 h). All KR-p110β-transfected cells were treated with tamoxifen (1 µM) for 12 h before analysis. Representative images **d**. and percentage of surviving cells **e**. analyzed as in **b**.. **f**. WB showing recombinant KR-PI3Kβ (examined with anti-myc Ab), pPKB, and controls; pPKB signal was quantitated as in **c**.. ** *P* < 0.01, *P < 0.05; Student's *t*-test.

We also analyzed the sensitivity of the UBC lines to a PI3Kβ inhibitor, AZD8186 [20]. 639V cells responded to AZD8186 in a dose-dependent manner (72 h), but cell death was lower than that detected upon si*PIK3CB* transfection, examined in parallel; both si*PIK3CB* and AZD8186 moderately reduced pPKB levels (Figure 3a-3c). Analysis of the remaining lines (Supplementary Figure S3a-S3c) showed that AZD8186 did not impair the growth of WT *PTEN* cells; in mutant*-PTEN* lines (UMUC-3, T24, J82), AZD8186 was less potent than si*PIK3CB* in reducing cell numbers; the si*PIK3CB*-resistant UMUC-9 and -7 lines were also resistant to PI3Kβ inhibitors; responsive cells showed moderate reduction of pPKB levels.

To confirm that a PI3Kβ scaffold function is more important than its kinase activity for UBC cell survival, we prepared an inactive si*PIK3CB*-resistant mutant KR-PI3Kβ (KR-PI3Kβ-si-res) that was not depleted by si*PIK3CB* (Figure 3d-3f). Although KR-PI3Kβ expression reduced pPKB more than si*PIK3CB;* KR- and KR-PI3Kβ-si-res reduced viability moderately while PI3Kβ depletion had a greater effect that was corrected by KR-PI3K-si-res expression (Figure 3d-3f). Thus, a PI3Kβ kinase-independent action contributes to cell survival in *PTEN*-mutant UBC.

### PI3Kβ depletion induces tumor regression in a *PTEN*-mutant UBC

**Figure 4:**
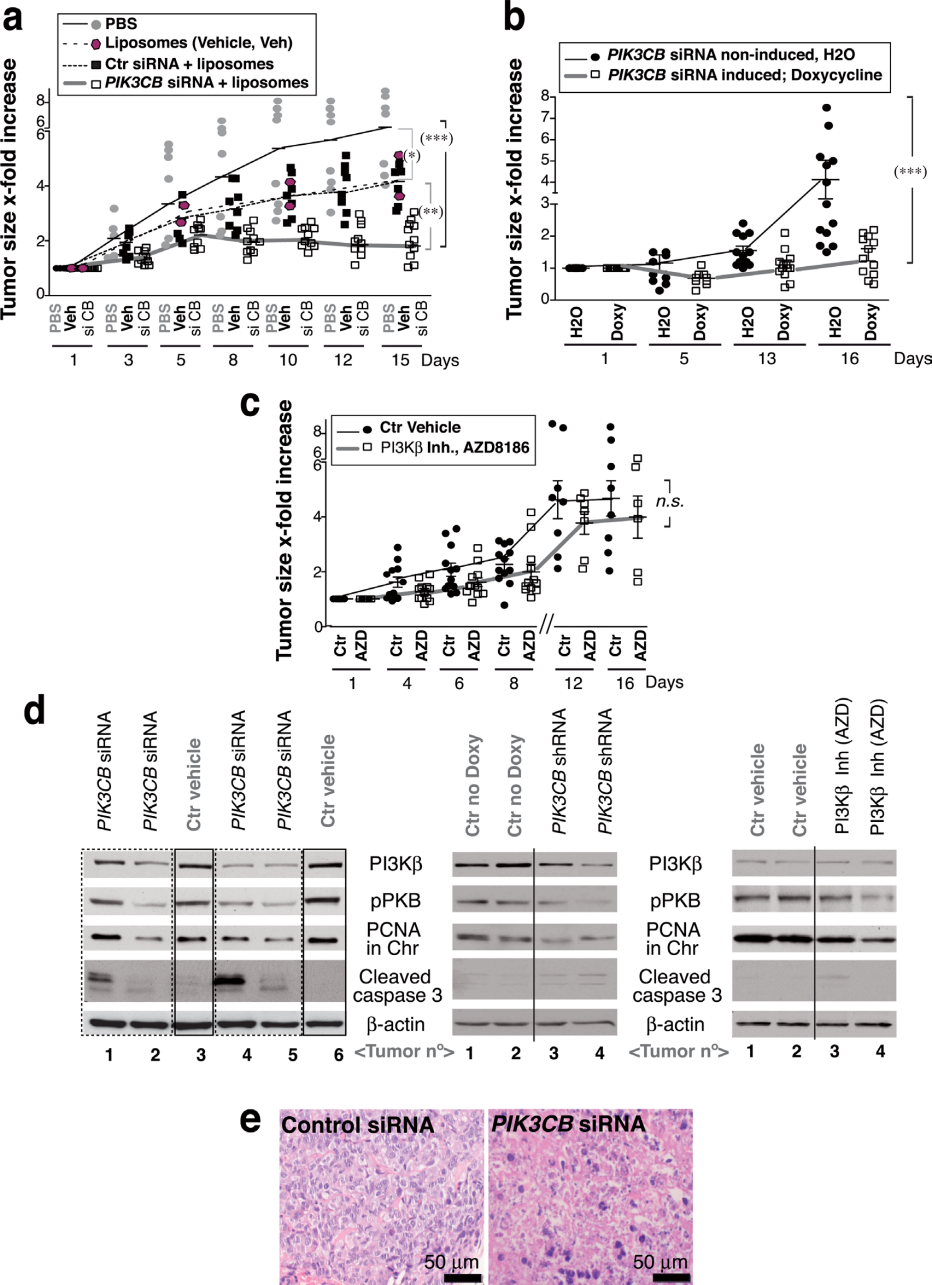
***PTEN***-negative, E-cadherin-low UBC cells are sensitive to ***PIK3CB*** depletion. **a**. To generate xenografts, 639V cells were injected subcutaneously into immunodeficient mice. When tumors reached 75 mm^3^, mice were treated by i.t. injection of PBS (control), vehicle alone (Invivofectamine, liposomes), or vehicle plus control or si*PIK3CB* (50pmol/mm^3^), every other day for two weeks. **b**. Xenografts were generated as in **a**. using 639V cells expressing doxycycline (Doxy)-inducible *PIK3CB* shRNA*.* When tumors reached ~75 mm^3^, ~half of the mice were treated by addition of Doxy (4 mg/ml) in the drinking water. **c**. Another set of tumors was generated similarly and was treated by i.p. injection of AZD8186 (40 mg/kg) in vehicle (methylcellulose) or with vehicle alone (control) every 12 h (days 1 to 5 each week) and once a day (days 6 and 7) for two weeks. (a-c) Graphs show the *x-*fold increase in tumor size (mean ± SD) at various times relative to volume at treatment initiation (day 1). *** *P* < 0.001; ** *P* < 0.01; * *P* < 0.05; *n.s*. not significant; one-way ANOVA, Tukey's comparison test. **d**. WB analysis of extracts from representative tumors (a-c). **e**. Hematoxylin/eosin staining of representative tumors from **a**..

To determine whether UBC also require PI3Kβ for tumor growth* in vivo*, we treated 639V xenografts with si*PIK3CB*. Tumors grew exponentially and when they reached ~75 mm^3^, they were treated by i.t. injection of PBS, liposomes, or liposomes plus control or si*PIK3CB* (50pmol/mm^3^). Although liposomes decelerated tumor growth, si*PIK3CB* was able to induce a partial tumor regression (Figure 4a). Considering that liposomes decelerated tumor growth, to confirm the effect of depleting *PIK3CB* in an alternative method, we generated tumors using 639V cells that expressed doxy-inducible sh*PIK3CB.* When tumors were ~75 mm^3^, mice were treated by addition of doxy in the drinking water (4 mg/ml) for two weeks. PI3Kβ depletion was partial (see below); nonetheless, tumors from untreated mice grew exponentially, while tumors from doxy-treated mice failed to grow or reduced its size (~50%) (Figure 4b). We also treated the tumors by i.p. injection with the PI3Kβ inhibitor AZD8186 [20] (40 mg/kg, every 12 h, 16 days). Pharmacological inhibition only moderately decelerated tumor growth (Figure 4c) and was associated with greater toxicity as 20% of treated mice had to be sacrificed prematurely due to weight loss.

PI3Kβ depletion in the tumors induced caspase 3 cleavage, reduced PI3Kβ expression and decreased PCNA levels in chromatin; it also slightly lowered pPKB levels; AZD8186 treatment reduced pPKB and PCNA chromatin binding and had a low effect on caspase 3 (Figure 4d). *PIK3CB* shRNA only partially depleted PI3Kβ; optimal *PIK3CB* depletion might have yielded additional effects. Histopathology analysis showed a moderate inflammatory infiltration in liposome-treated samples, few necrotic cells in controls and a large percentage of dead cells in si*PIK3CB*-treated tumors (50-100%) (Figure 4e). This analysis shows that PI3Kβ depletion reduced UBC tumor growth more markedly than PI3Kβ inhibition.

### E-cad/N-cad expression determines *PTEN*-mutant UBC dependence on PI3Kβ

**Figure 5:**
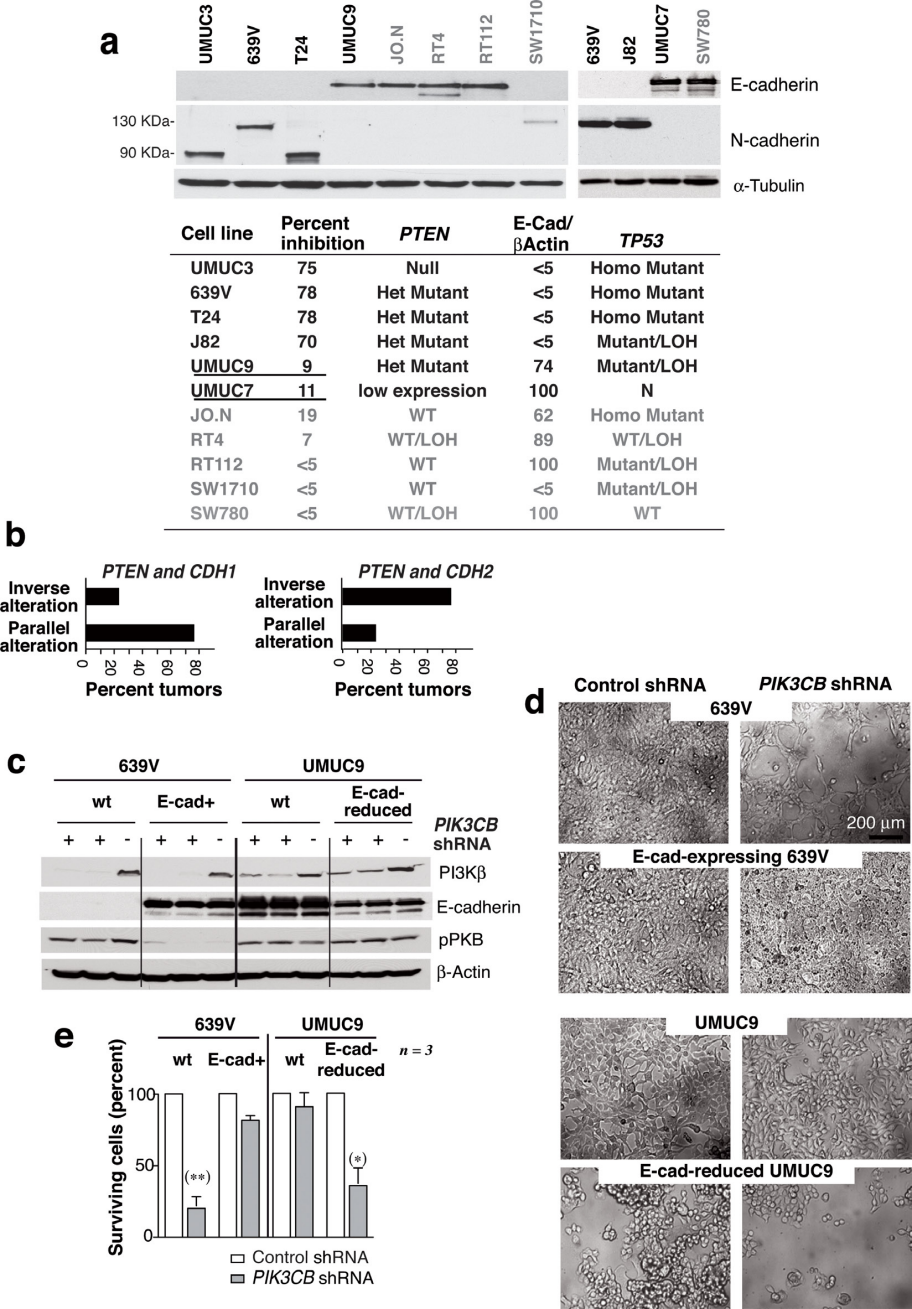
E-cadherin expression determines UBC line sensitivity to si ***PIK3CB***. **a**. E-cad and N-cad levels in UBC cell line extracts were examined in WB. The table summarizes the E-cad/β-actin signal ratio relative to maximal levels (100%), the *TP53* and *PTEN* mutational status, and the UBC cell responses to PI3Kβ silencing expressed as a percentage of cell survival inhibition (shown in Figure 2). Mutant *PTEN,* E-cad-high expressing cells are underlined. Het, heterozygous; Homo, homozygous; LOH, loss of heterozygosity; N, normal copy number. **b**. Graphs show UBC (from TCGA) with *PTEN* and *CDH1* (E-cad) alterations or with *PTEN* and *CDH2* (N-cad) alterations classified as tumors with parallel or inverse alterations in these genes (see Suppl. Figure S4). (c-e) 639V cells and 639V clones expressing E-cad were transfected with cDNA encoding doxy-inducible-control or* -PIK3CB* shRNA. Control and E-cad-depleted UMUC-9 cells were also transfected with control or inducible-*PIK3CB* shRNA. Extracts were tested in WB after doxy induction (5 µg/ml, 96 h) **c**.; images show representative fields **d**.. **e**. Percentage of cells after *PIK3CB* depletion (96 h) relative to controls. ** Student's *t*-test* P* < 0.01, *P < 0.05.

PI3Kβ depletion in UBC showed that *PTEN* loss of function is necessary but not sufficient for PI3Kβ dependence. We compared the *TP53* mutation status [33] and E-cad levels in the UBC lines. Of the six lines exhibiting *PTEN* loss of function, the four PI3Kβ*-*dependent cells had little or no E-cad, and expressed 90 or 130 kDa N-cad forms; in contrast, the si*PIK3CB-*resistant cells (UMUC-9 and -7) expressed high E-cad levels (Figure 5a), suggesting that also in UBC low E-cad expression is needed for PI3Kβ dependence.

*Pten* loss of function cooperates with *Lkb1* or *Tp53* mutations to initiate EMT, which is associated with E-cad loss and N-cad expression [29, 34]. Using the Cancer Genome Atlas UBC tumor collection, we evaluated whether* PTEN* loss of function was linked to E-cad (*CDH1*) downregulation or N-cad (*CDH2*) expression. *PTEN* alterations in UBC were associated with *CDH2* upregulation (*P* = 0.01, www.cbioportal.org) (Supplementary Figure S4). Of the samples with altered *PTEN* and *CDH1*, 75% showed parallel gain or loss of function in both genes; in contrast*,* 75% of samples with alterations in* PTEN* and* CDH2* showed an inverse pattern of alterations in both genes (Figure 5b). This suggests that *PTEN* loss of function is associated to E-cad loss and N-cad upregulation in UBC.

The association of* PTEN* loss of function with *CDH2* upregulation made difficult to find mutant *PTEN* UBC lines that expressed E-cad (two in the UBC collection; two in the diverse tumor set). To explore the hypothesis that low E-cad levels and *PTEN* mutation are both necessary for UBC dependence on PI3Kβ, we used a genetic approach and examined the effect of E-cad reconstitution in a responsive cell line lacking E-cad expression (639V) or its depletion in an E-cad-expressing resistant cell line (UMUC-9). E-cad reconstitution rendered 639V cells resistant to* PIK3CB* shRNA, whereas E-cad reduction yielded UMUC-9 cells sensitive to *PIK3CB* depletion (Figure 5c-5e). pPKB levels were moderately reduced by PI3Kβ depletion in E-cad-low 639V cells, but pPKB was unaffected by PI3Kβ depletion in WT or E-cad-depleted UMUC-9 cells (Figure 5c). We confirmed that *PIK3CB* depletion induced apoptosis in E-cad-depleted but not in control UMUC-9 cells (Supplementary Figure S5).

### E-cadherin depletion sensitizes *PTEN*-mutant UBC tumors to *PIK3CB* silencing *in vivo*

**Figure 6:**
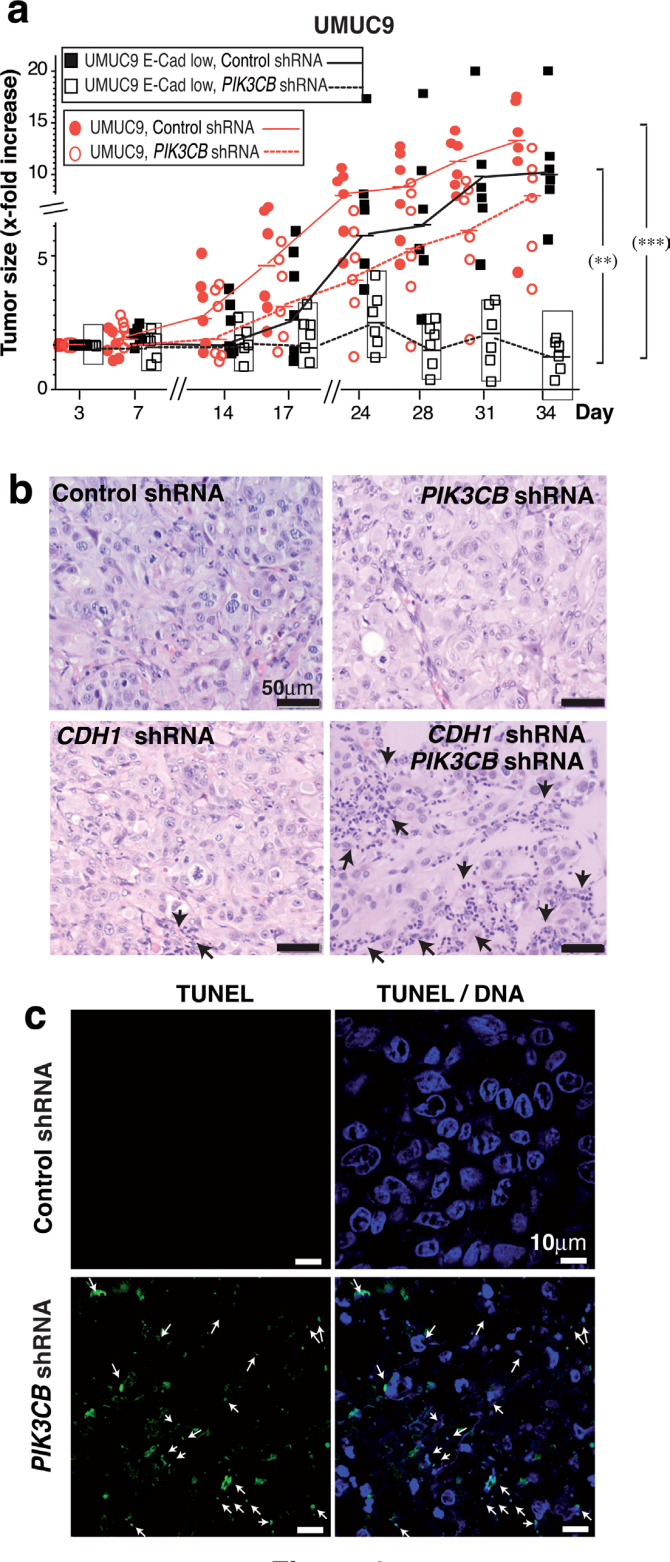
E-cadherin depletion sensitizes ***PTEN***mutant UBC cells for ***PIK3CB*** silencing. **a**. Xenografts were generated by injecting E-cad-depleted UMUC-9 cells (5 x 10^6^) in PBS:Matrigel (1:1) into both flanks of immunodeficient mice. When most tumors reached ~75 mm^3^, doxycycline was added to drinking water and tumor size measured every 3 days. The graph shows the *x-*fold increase in tumor size (mean ± SD) at different times relative to the volume at treatment initiation (day 1). *** *P* < 0.001; ** *P* < 0.01; one-way ANOVA Tukey's multiple comparison test. (b, c) Representative histochemistry **b**. and TUNEL analysis **c**. of indicated tumor sections.

To confirm the sensitivity of control and E-cad-low UMUC-9 cells to *PIK3CB* silencing *in vivo*, we used UMUC-9 cells stably expressing *CDH1* shRNA and doxy-inducible *PIK3CB* shRNA. When xenografts reached ~75 mm^3^, we administered doxycycline. *PIK3CB*-depletion moderately affected UMUC-9 xenografts growth; moreover, *PIK3CB*-depletion induced regression of E-cad-low UMUC-9 tumors (Figure 6a). Histopathology analysis of representative tumors at assay termination showed that, at difference from controls, tumor vestiges from E-cad-low *PIK3CB* shRNA-treated UMUC-9 cells were composed essentially of dying or dead cells; TUNEL analysis of these samples confirmed apoptosis in *PIK3CB* shRNA-treated tumors from E-cad-low UMUC-9 cells (Figure 6b, 6c).

### PI3Kβ controls N-cadherin adhesions

E-cad-negative UBC form N-cad adhesions [35, 36] that are essential regulators of survival [37; we showed that *PTEN* loss of function was frequently linked with N-cad increase in UBC. Since E-cad loss and the accompanying increase in N-cad levels are needed for PI3Kβ dependence in UCB, we postulated that PI3Kβ might regulate N-cad adhesions. We treated PI3Kβ-dependent (639V or J82) or PI3Kβ-independent UBC cells (SW780 or RT112) (0.5x10^6^) with AZD8186 for three days, or transfected them with si*PIK3CB1* (as above) and we examined cell-cell adhesions by immunofluorescence (IF).

**Figure 7:**
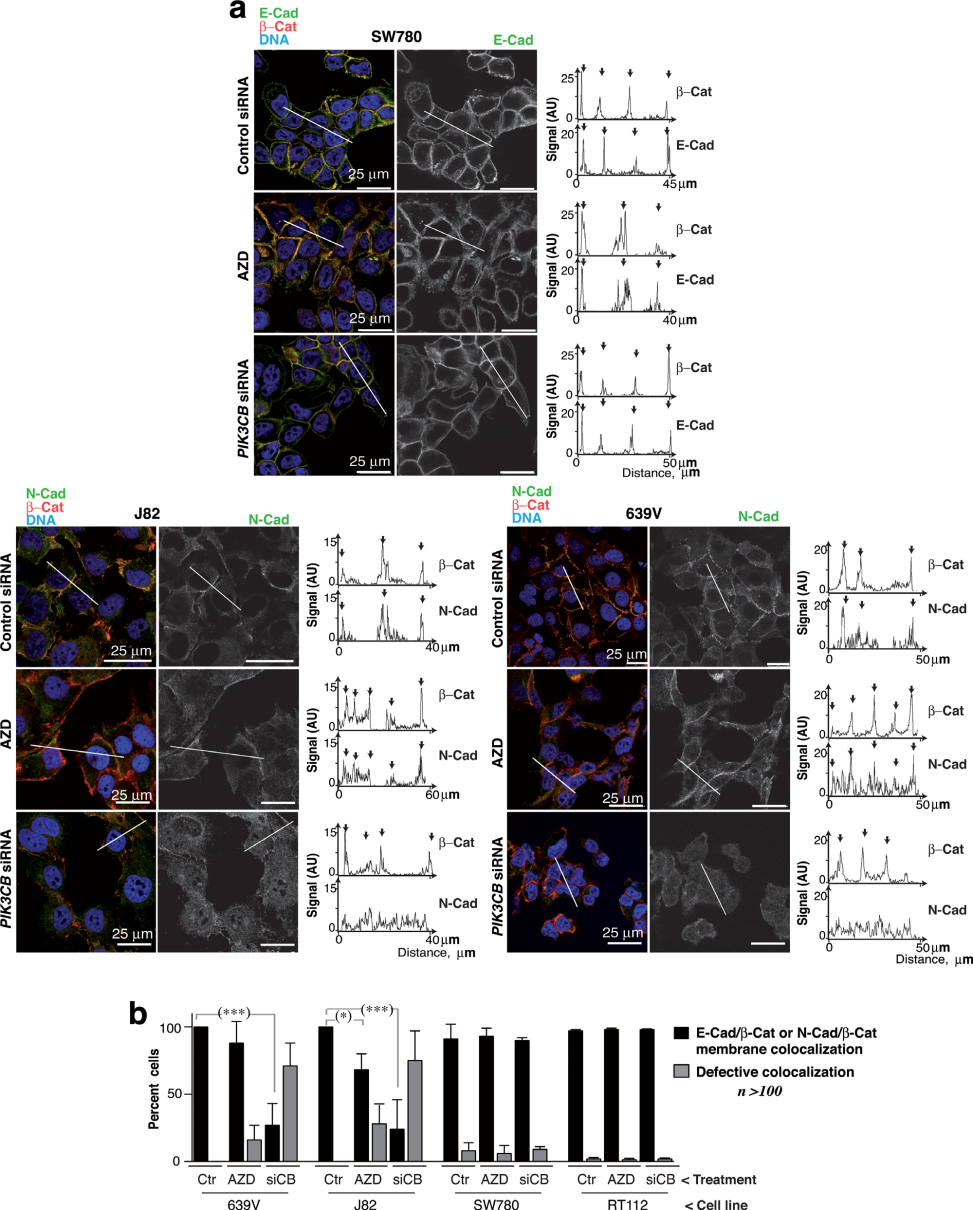
PI3Kβ depletion selectively impairs N-cadherin-mediated cell adhesions **a**. 639V, J82 and SW780 cells were transfected with control or si*PIK3CB1* shRNA or cultured with 250 nM AZD8186 (72 h). Cells were stained with the indicated antibodies and examined by confocal microscopy (indicated). Representative z-sections are shown. Quantitation of N-cad or β-cat signal in arbitrary units (AU) in the linear plots (indicated). **b**. Percentage of 639V, J82, RT112 and SW780 cells with normal cell adhesions defined as those showing N-cad (or E-cad) and β-cat colocalization in the membrane region between adjacent cells in at least in one side of the cell. *** *P* < 0.001, * *P* < 0.05; Student's *t*-test.

E-cad adhesions in SW780 cells were virtually unaffected by PI3Kβ depletion or inhibition. E-cad and β-catenin (β-cat) colocalized at cell-cell interaction regions similarly in controls than in cells treated with AZD8186 or with si*PIK3CB1*, as examined by E-cad/β-cat signal quantitation at cell membranes (Figure 7a). We also detected N-cad/β-cat colocalization at plasma membranes of 639V and J82 cells treated with control siRNA or AZD8186; in contrast, PI3Kβ depletion severely disrupted N-cad localization at cell-cell adhesions (Figure 7a). Examination of the percentage of cells exhibiting N-cad (or E-cad) colocalization with β-cat at cell-cell adhesions confirmed that PI3Kβ did not affect E-cad adhesions but impaired N-cad localization in ~75% of 639V and J82 cells (Figure 7b).

To test whether interference of N-cad adhesions could promote cell death, we used interfering peptides for N-cad and E-cad in 639V and J82 cells; incubation with specific peptides for N-cad reduced cell viability in 639V and J82 cells (Supplementary Figure S6). *PTEN* alterations in UBC associate with N-cad upregulation; our findings suggest that PI3Kβ controls cell survival in these UBC by regulating N-cad adhesion integrity.

### PI3Kβ associates with N-cadherin

**Figure 8:**
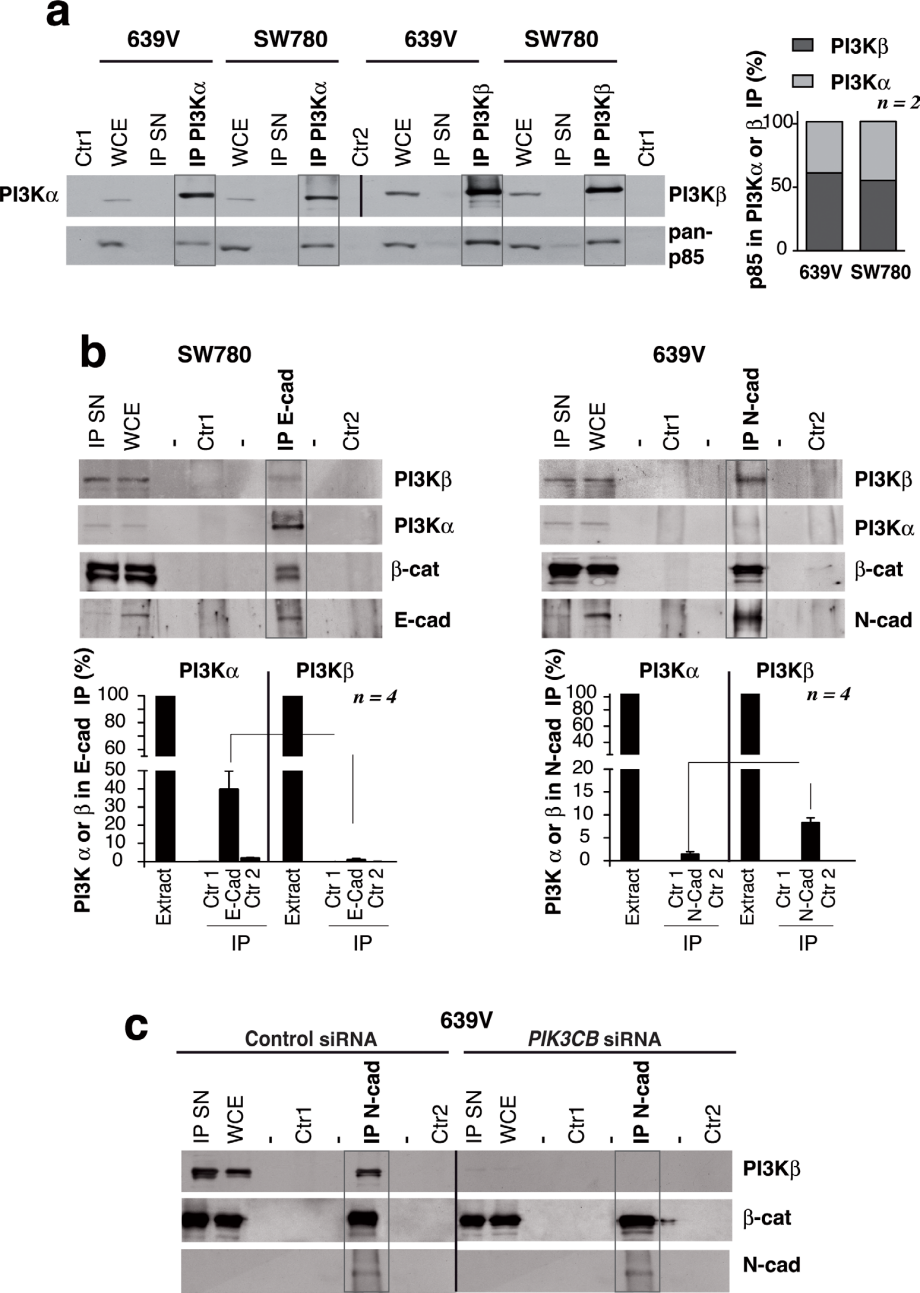
PI3Kβ associates with N-cadherin in ***PTEN*** mutant, E-cadherin-low UBC cells. **a**. Total extracts from 639V and SW780 cells (50 µg) or immunoprecipitates (IP) from these extracts (1 mg) were prepared with anti-PI3Kα or anti-PI3Kβ Ab and were tested in WB. PI3Kα IP and its controls were tested using PI3Kα Ab; PI3Kβ IP and its controls were blotted with PI3K Ab; all the samples were simultaneously examined with a pan-p85 Ab. Control 1 (Ctr1), non-specific signal in immunoprecipitates without antibody; Control 2 (Ctr2), lysis buffer incubated with antibody; IP SN, supernatant of IP (~30 µg). WB using pan-p85 Ab showed that 639V and SW780 cells had similar PI3Kα /PI3Kβ proportion as form both we isolated moderately higher amounts of p85/PI3Kβ than p85/PI3Kα complexes. The graph represents the percent of p85 signal (mean SEM) associated to PI3Kα or PI3Kβ referred to total p85 signal in both IP (100%) in each cell line. **b**. Extracts (900 µg) from 639V or SW780 cells were immunoprecipitated using N-cad or E-cad Ab, respectively; complexes were analyzed in WB. Total cell extracts (30 µg) were resolved in parallel. We quantitated the PI3Kα signal per µg in WCE (considered 100%) as well as PI3Kα signal per µg in the IP that was referred to maximal. We performed a similar quantitation of PI3Kβ signal. Graphs show the percent of PI3Kα (or of PI3Kβ signal) associated to cadherins in 639V or SW780 cells (mean SEM, *n = 4*). Controls as in **a**.. **c**. Specificity of the PI3Kβ band in complex with N-cad was tested as in **b**. using control or PI3Kβ-depleted 639V cell extracts. Controls as in **a**.. **P < 0.001, *P < 0.05; Student's *t*-test.

PI3Kβ expression is needed for N-cad adhesions integrity; we tested whether PI3Kβ could bind to N-cad and thereby exert a scaffold function for N-cad membrane localization. We first examined PI3Kα and PI3Kβ levels in E-cad+ SW780 cells and N-cad+ 639V cells. To determine the PI3Kα / PI3Kβ ratios in the cells, we immunoprecipitated PI3Kα and PI3Kβ from extracts of SW780 and 639V cells (900 µg). As PI3K associates 1:1 with p85, we compared the amount of p85/PI3Kα and p85/PI3Kβ complexes by examining in WB the associated p85 subunit. To check that the immunoprecipitation (IP) was efficient we confirmed that the supernatants of the IP were depleted of the PI3Kα or β; both cell lines showed moderately higher levels of p85/PI3Kβ than p85/PI3Kα (Figure 8a). E-cad IP from SW780 cell extracts followed by WB with PI3Kα or PI3Kβ Ab showed that E-cad preferentially associates PI3Kα (Figure 8b). A similar analysis after N-cad IP from 639V extracts showed that N-cad preferentially associates PI3Kβ (Figure 8b). For quantitation, we referred the amount of PI3Kα or β associated to N-cad or E-cad to the total PI3Kα or β cell content (in cell extracts). We confirmed that the PI3Kβ signal in complex with N-cad was specific, since *PIK3CB* depletion abolished this signal (Figure 8c).

## Discussion

There is increasing evidence that inhibition of oncogenic kinases activities does not equate to the reduction of their expression. This is the case of PI3Kβ, whose expression, rather than its activity, is necessary from the onset of embryonic development [24-26]. We explored the therapeutic potential of local *PIK3CB* siRNA delivery on* PTEN*-mutant tumors, since PI3Kβ is the main isoform activated in these tumors [15]. After examining a set of 10 unrelated cancer cell lines, we focused on a panel of 11 bladder carcinoma cells (UBC), as these tumors have *PTEN* mutations and increased PI3Kβ expression. In most *PTEN*-mutant UBC cells, *PIK3CB* knockdown induced apoptosis and effectively led to tumor regression; PI3Kβ depletion was markedly more efficient than PI3Kβ inhibition. The apoptotic response was absent in a few samples, which provided an opportunity to determine the molecular requirements for PI3Kβ dependence. Our results suggest that *PTEN* mutation and concomitant E-cad loss (found in invasive UBC) are needed for PI3Kβ dependence. In parallel to E-cad loss, UBC express N-cad [35], which controls cell survival [38]. We show that PI3Kβ associates with and regulates N-cad adhesion.

Approximately half of human cancers have mutations in PI3K or in its negative regulator *PTEN*; the latter are more frequent at advanced tumor phases [3, 4, 39]. PI3K inhibitors have been tested clinically; some of these studies hold promise, but others have failed due to blockade of PI3K feedback pathway regulation or generation of resistance [21-23, 40-42], making necessary the development of additional therapeutic approaches. As proof of concept for siRNA-based local treatment of UBC, we optimized i.t. injection of siRNA in UBC xenografts. Local siRNA delivery is anticipated to reduce dose requirements and minimize toxicity [43, 44]. i.t. siRNA delivery reduced xenograft size, triggered apoptosis, and was more effective than PI3Kβ inhibition, which supports siRNA injection as a feasible approach. The vehicle used, however, showed partial toxicity, been necessary development of optimal siRNA vehicles. Since all vehicle- (liposome) treated samples showed an inflammatory neutrophil infiltration, it is possible that an action of *PIK3CB* siRNA in the tumor environment contributes to tumor growth deceleration.

Local delivery appears to be suitable for tumors with increased *PIK3CB* expression that are accessible to local administration, such as bladder cancer. Indeed, high-risk non-MI UBC is currently treated by intravesical therapy with Bacille-Calmette Guerin [7], and si*PIK3CB* treatment can be reduced to two injections a week for two weeks; intravesical infusion with a defective virus encoding* PIK3CB* shRNA or the use of novel non-toxic vehicles, currently under development [45], might be suitable reagents for *PIK3CB* depletion. Several siRNA targets are currently in clinical trials, and the large number of successful phase II and III trials for human disease predicts the incorporation of these therapies for medical applications [46, 28]. Development of antisense oligonucleotides [47] or application of the successful CRISPR-Cas approach [48] for* PIK3CB* deletion might also be useful for local treatment of advanced UBC (T2b and beyond) bearing *PTEN* mutations.

PI3Kβ depletion was more effective than its inhibition in reducing UBC tumor growth; whereas the inhibitor AZD8186 decelerated tumor expansion, PI3Kβ depletion induced caspase 3 cleavage and tumor regression. The observation that PI3Kβ has both enzymatic and non-enzymatic functions [24-26] might explain the effectiveness of si*PIK3CB* in the *PTEN*-mutant tumors. We tested the contribution of the PI3Kβ scaffold function in several ways. We compared the effect of depleting or inhibiting PI3Kβ in the growth of UBC lines in culture. *PTEN* loss of function was necessary for sensitivity to PI3Kβ inhibition or depletion, however, cell numbers were moderately reduced by inhibitor and markedly reduced by PI3Kβ depletion. pPKB levels were unchanged or only modestly diminished by PI3Kβ depletion. Moreover, whereas expression of an inactive KR-PI3Kβ reduced 639V cell viability moderately, PI3Kβ depletion had a greater effect on cell death induction, which was reduced by co-expression of a si*PIK3CB*-resistant inactive PI3Kβ mutant. We also found that PI3Kβ depletion has a more potent effect than its inhibition on disruption of N-cad adhesions. These observations support a PI3Kβ scaffold function in UBC survival.

A small proportion of* PTEN*-defective cells were resistant to* PIK3CB* silencing; we evaluated the molecular characteristics of these cell lines. The all four si*PIK3CB*-resistant *PTEN*-mutant cells (two UBC and two from the diverse tumor set) exhibited had high E-cad levels. Invasive UBC show *PTEN* defects (58% of tumors) [9, 10, 39], *PIK3CB* upregulation, and mesenchymal phenotypes [35, 49-51]. In the mouse, *Pten* loss of function cooperates with *Lkb1* or *Tp53* mutation to initiate EMT, which induces E-cad downregulation and N-cad expression [29, 34-36]. We examined the Cancer Genome Atlas bladder cancer collection ( www.cancergenome.nih.gov) to evaluate the possible linkage of *PTEN* mutation and E-cad or N-cad expression in UBC. Simultaneous *TP53* and *PTEN* mutation did not correlate with changes in E-cad or N-cad expression, nonetheless, *PTEN* alterations in UBC were associated with *CDH2* upregulation (*P* = 0.01, www.cbioportal.org) supporting a link between *PTEN* and E-cad/N-cad expression changes. Moreover, using a genetic approach, we found that E-cad-expression in 639V-PI3Kβ-dependent cells made them resistant to PI3Kβ depletion while E-cad silencing sensitized UMUC-9 cells to* PIK3CB* depletion. This suggests that si*PIK3CB* resistance in *PTEN* mutant UBC is due to E-cad expression.

si*PIK3CB*-sensitive and insensitive lines can be distinguished by E-cad/N-cad levels; we studied the consequences of depleting or inhibiting PI3Kβ on cell-cell adhesions. We used two E-cad-high (PI3Kβ*-*insensitive) and two E-cad-low/N-cad-positive (PI3Kβ-sensitive) UBC lines. In insensitive lines, cell adhesions were unaffected by PI3Kβ depletion or inhibition; in sensitive lines, PI3Kβ inhibition moderately reduced, but PI3Kβ depletion abrogated, N-cad-adhesions in more than 75% of the cells. PI3Kβ expression thus regulates N-cad adhesions in UBC. We show that E-cad bound preferentially PI3Kα in *PTEN-*WT SW780 cells*;* in *PTEN-*mutant 639V cells, N-cad bound to preferentially PI3Kβ*.* As cell adhesions are needed for UBC cell survival (Figure S6), these results suggest that PI3Kβ exhibits a scaffold function for N-cad adhesions stabilization, which regulate UBC survival.

Acquisition of resistance in 639V cells after E-cad expression can be explained by considering that PI3Kβ depletion does not affect E-cad adhesions. We also show that E-cad reduction in UMUC-9 cells renders them sensitive to PI3Kβ depletion although these cells do not express N-cad. The reduction in E-cad levels and an additional survival effect mediated by PI3Kβ (such as Bim regulation, data not shown) might explain their sensitivity to *PIK3CB* depletion.

*PTEN* alterations in UBC are associated with *CDH2* upregulation. In these cells, PI3Kβ associates with and is needed to maintain N-cad cell adhesions. As these adhesions regulate cell survival in UBC, disruption of N-cad adhesions is at least part of the mechanism for the greater cytotoxic effect of PI3Kβ depletion compared to PI3Kβ inhibition in *PTEN* mutant UBC. There is increasing evidence that high-risk non-MI bladder tumors are precursors of MI cancers, with whom they share genomic features [7]. High-risk tumors confined to the bladder are generally treated by intravesical therapy, but up to 30% progress to muscle-invasive UBC, which have poor prognosis and often develop metastases. Our results support the use of strategies that decrease *PIK3CB* expression as a treatment for high-risk non-muscle-invasive bladder cancer with *PTEN* mutation and low E-cad levels, a frequent phenotype in advanced urothelial carcinoma.

## Materials and Methods

### Antibodies, reagents, siRNA, shRNA and cDNA

Antibodies used were anti-p110β, -p110α and -pPKB (Ser 473) (Cell Signaling), pan-p85 (Millipore), -E-cad, PCNA (BD Biosciences), -PKB (Upstate Biotechnology), -β-actin, -α-tubulin (Sigma), -N-cad (Abcam) and --catenin (BD). For *in vivo* assays, we used Stealth *PIK3CB* siRNA that targeted the sequences AACCACTGGAATTTGATATTAATAT (siRNA1) or GATTCACAGATAGCATCTGAT (siRNA2), or control (scrambled-siRNA1) and vehicle (Invivofectamine), (Invitrogen). We prepared doxycycline (doxy)-inducible-pLKO-tet-*PIK3CB* shRNA vector by subcloning in the EcoRI site the oligonucleotide CCGGGATTCACAGATAGCATC TGATCTCGAGATCAGATGCTATCTGTGAATCTTTTT and its reverse complementary oligonucleotide. We used RNAiMAX (Invitrogen) and OptiMem (Life Technologies) for siRNA transfection. pLKO-Puro-*CDH1* shRNA and the dexamethasone (Dex)-inducible pLK-pac vector encoding *CDH1* have been described [52]. The PI3Kβ inhibitor AZD8186 was from Astra Zeneca [20] . PSG5-ER-myc-K805R-p110β was prepared as described [25]. To generate a si*PIK3CB* resistant-K805R-p110β, we used the oligonucleotide GGAATGA ACCACTCGAATTTCATATTAATATTTGTGACTT ACCAAGAATGG and its reverse complementary in a PCR reaction using Quickchange (Agilent). ER-myc-KR-p110β was cotransfected with siRNA using Lipofectamine 2000 (Invitrogen); these cells were treated with tamoxifen (1 µM, Sigma) 12 h before analysis.

### Cell lines

We used the cell lines PC3 (ATCC-CRL-1435), U87MG (ATCC-HTB-14), BLM (lung metastasis of BRO melanoma cells, confirmed by typing the short tandem repeat DNA profiles at Genomics Service, Instituto de Investigaciones Biomédicas Alberto Sols, Madrid), HT1080, HT-29, SW680, MDA-MB468 (ATCC-CCL121, -HTB38, -228 and HTB-132, respectively), and NCI-H226, -H520 and CaLu-1 (ATCC-CRL-5826, -HTB182 and HTB-54, respectively). We also used 639V, UMUC-3, UMUC-7, UMUC-9, J82, T24, RT4, RT112, JON, SW1710 and SW780 urethral bladder carcinomas [33]. Cells were maintained in DMEM or RPMI (Gibco-BRL) with 10%fetal bovine serum, 2mM glutamine, 10mM HEPES, 100 U/ml penicillin, and 100µg/ml streptomycin (37ºC, 5%CO_2_). We prepared UMUC-9 cell clones expressing shRNA for *CDH1* using lentiviral particles containing the pLKO-Puro-*CDH1* shRNA. Clones were selected in medium with puromycin (1-2µg/mL). We similarly generated 693V lines overexpressing E-cad using lentiviral particles containing the Dex-inducible pLK-pac-*CDH1* cDNA. Stable clones were selected with puromycin (5µg/mL); E-cad expression was induced in medium with 10 nM Dex (48 h). Clones expressing pLKO-tet-*PIK3CB*-shRNA vector (doxy-inducible) were prepared similarly and were selected in medium containing G418 (0.8 mg/ml, 10 days).

### Mice wearing xenografts treatment

All procedures using mice were approved by the Ethics Committee of the CNB/CSIC in accordance with EU/Spanish legislation (RD53/2013). To generate xenografts, cells (2.5-to-5x10^6^) were suspended in 100 µl PBS containing 25% (639V and PC3 cells) or 50% Matrigel (UMUC-9 cells) and were injected subcutaneously (s.c.) into both flanks of 2-to-3-month-old immunodeficient CB17/lcrHsd-*Prkdc*scid*/Lys* bg-j mice (Harlan). When tumors reached a mean size of 75 mm^3^, mice were treated by direct central intratumor injection (Hamilton syringe 702N, 25 µl, 22-g bevel tip needle) with vehicle, PBS alone, or either control or si*PIK3CB* in Invivofectamine. si*PIK3CB* was dissolved in PBS, followed by Invivofectamine (0.8:1 Invivofectamine:siRNA in PBS). siRNA doses were 12.5, 50 or 125 pmol/mm^3^ of tumor volume. For optimal *PIK3CB* depletion, 50 pmol per mm^3^ of tumor volume and dose si*PIK3CB1* was sufficient and was used for subsequent experiments. Mice were treated three times weekly for two weeks, although two injections in two weeks were similarly efficient.

When tumors of cells that expressed inducible sh*PIK3CB* reached ~75 mm^3^, silencing was induced by doxy (4 mg/ml in H_2_O with 5% glucose) to drinking water. Tumor-bearing mice also were treated by i.p. injection with the PI3Kβ inhibitor AZD8186 (40 mg/kg) in 0.5% methylcellulose, 0.2% Tween-20. Tumor size was measured with a caliper and volume was calculated as (V) = (smaller side length^2^ x larger side length)/2.

### TUNEL, annexin V, histology, western blot, flow cytometry, immunofluorescence, immunoprecipitation and peptide assays

TUNEL assays were performed using the DeadEnd Fluorometric TUNEL System Kit (Promega); the Annexin V-FITC assay was from Southern Biotech. Histology was as described [18]. Cell extracts were prepared in RIPA buffer as described [25]; WB and cell fractionation were as described [25]. Sub-G1 DNA-containing cells were tested by flow cytometry [25]. Immunofluorescence procedures were described [26]; for immunoprecipitation, cells were lysed in buffer containing 10 mM Tris-HCl pH7.4, 150 mM NaCl, 10 mM KCl, 1% NP-40 with phosphatase and protease inhibitors, and precipitated as reported [25]. We used three synthetic peptides bearing E-cad (LFSHAVSSNG) or N-cad (LRAHAVDING) sequences or a “scrambled” sequence with no known matches (ARLHDVAING). Peptides were N-acetylated and C-amidated (Biomatik); UBC cells were cultured with peptides (400 µg/ml) for different periods prior to analysis.

### Analysis of PIK3CB expression levels and of *PTEN*, *CDH1* and *CDH2* changes in UBC

We evaluated *PIK3CB* mRNA levels based on previously reported DNA microarray data. The first dataset (94 samples), includes 9 normal bladder biopsies, 56 NMI tumors and 29 MI tumors [30, 31] (GEO:GSE5287). The second dataset (44 tumor samples, includes 34 NMI and 8 MI tumors [32] (GEO:GSE71576). These analyses were performed using Affymetrix U133A:212688 and Affymetrix 1.0 ST 8091009 probe hybridization data. The third dataset was from ArrayExpress ( www.ebi.ac.uk/arrayexpress/) with accession numbers E-MTAB-1803 for MI-UBC and E-MTAB-1940 for non-MI-UBC. RMA software (v1.20) from Bioconductor was used to normalize gene expression with express function and parameters by default. We used the Cancer Genome Atlas [42] (TCGA) bladder cancer collection ( www.cancergenome.nih.gov) and the software at http://www.cbioportal.org/index.do to examine the relationship between* PTEN*, *CDH1* and *CDH2*.

### Statistical analyses

Gel band intensity was quantitated with ImageJ software (NIH). Significance was calculated (GraphPad Prism 5.0 Software) using one-way ANOVA followed by Tukey's multiple comparison test, Kruskal-Wallis, one- or two-tailed Mann-Whitney tests, and Student's *t*-tests.

## SUPPLEMENTARY MATERIALS FIGURES


