# Supplementary material for: E-cadherin downregulation sensitizes PTEN-mutant tumors to PI3Kβ silencing
Source: Oncotarget. 2016 Nov 16;7(51):84054–71. doi: 10.18632/oncotarget.13414 (PMC5356644; doi:10.18632/oncotarget.13414)
Supplement: Supplementary file 1 [file oncotarget-07-84054-s001.pdf]

E-cadherin downregulation sensitizes *PTEN*-mutant tumors to PI3Kβ silencing

Supplementary Material

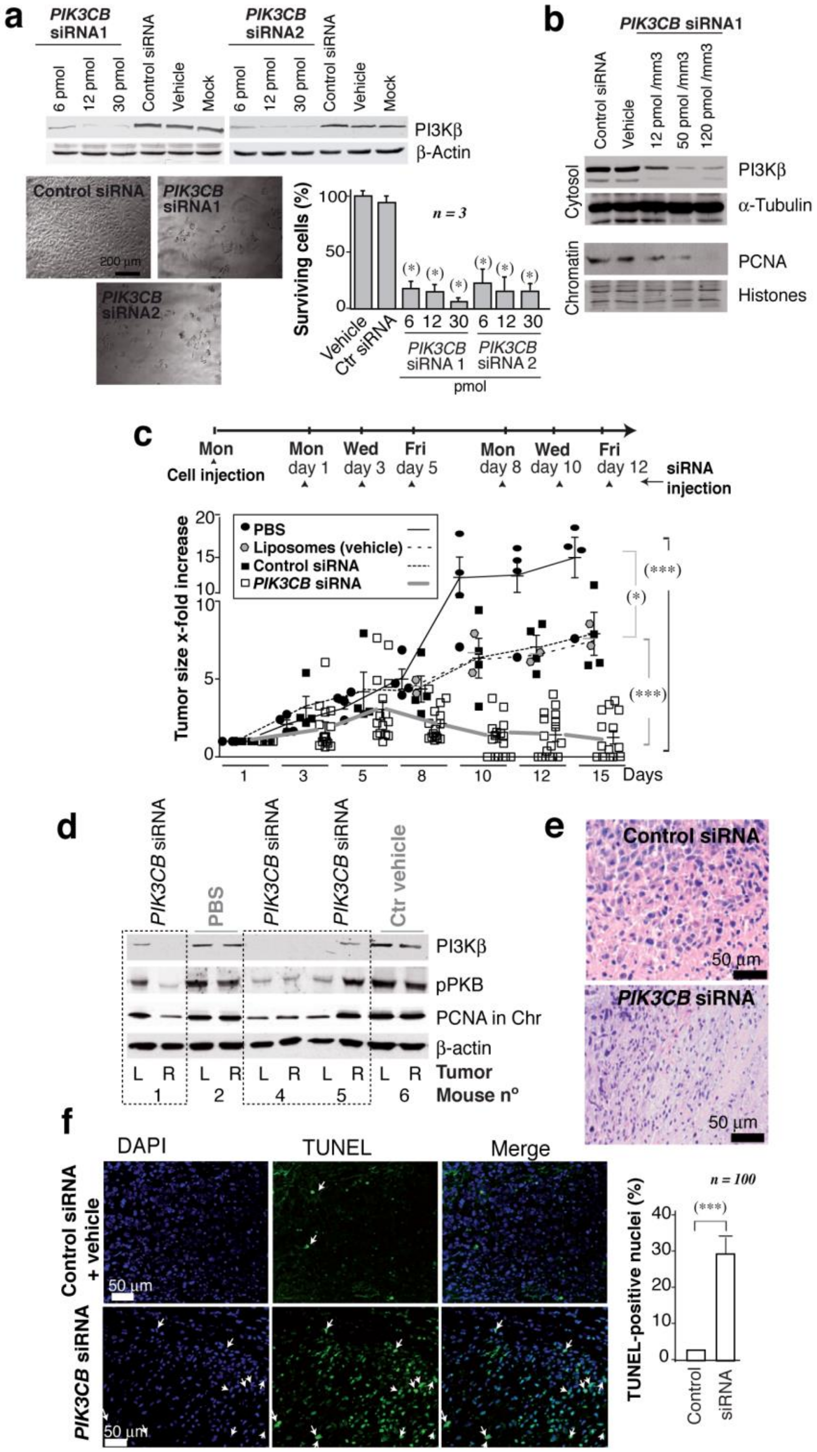

Suppl. Figure S1. Intra-tumor injection of PC3 human prostate xenografts with siPIK3CB triggers apoptosis.
(**a**) PC3 cells (2 x 10^5) were transfected with si*PIK3CB* (indicated doses) for 72 h. Effect on PI3Kβ expression as determined in WB, and the appearance of the PC3 cell cultures at 72 h post-transfection with control (scrambled), *PIK3CB* siRNA1 or siRNA2 (12 pmol/2 x 10^5 cells). Percentage of surviving cells at 72 h compared to time 0 (mean ± SD, n = 3). (**b**) To generate xenografts, cells (2.5 x 10^6 in 100 µl PBS plus 25% Matrigel) were injected s.c. into both flanks of immunodeficient mice. When tumors reached a mean size of ~75 mm3, mice were treated by i.t. injection with 12.5, 50 or 120 pmol/mm^3 tumor in vehicle. We administered si*PIK3CB1* on days 1, 3, 5; tumors were collected and tested in WB on day 7. Results were similar using si*PIK3CB2* (**c**) Regime for i.t. si*PIK3CB1* administration to xenograft tumors. Xenografts were generated as in (b); when most tumors reached ~75 mm3, mice were treated with an i.t. injection of PBS, vehicle alone (liposomes), or 50pmol/mm^3 control or si*PIK3CB1* in vehicle, three times a week for two weeks. The graph shows the x-fold increase in tumor size (mean ± SD) compared to size at initiation of treatment (day 1). (**d**) WB analysis of tumor extracts from (c) using indicated antibodies. *** $P < 0.001$; ** $P < 0.01$; * $P < 0.05$; one-way ANOVA followed by Tukey's multiple comparison test. (**e, f**) Hematoxylin/eosin (e) and TUNEL staining (f) of representative tumor sections from (c). The graph (f) shows the percentage of TUNEL-positive nuclei in sections of control and siPIK3CB-treated mice tumors (mean ± SD). (a, f) * $P < 0.05$; *** $P < 0.001$; Student's t-test.

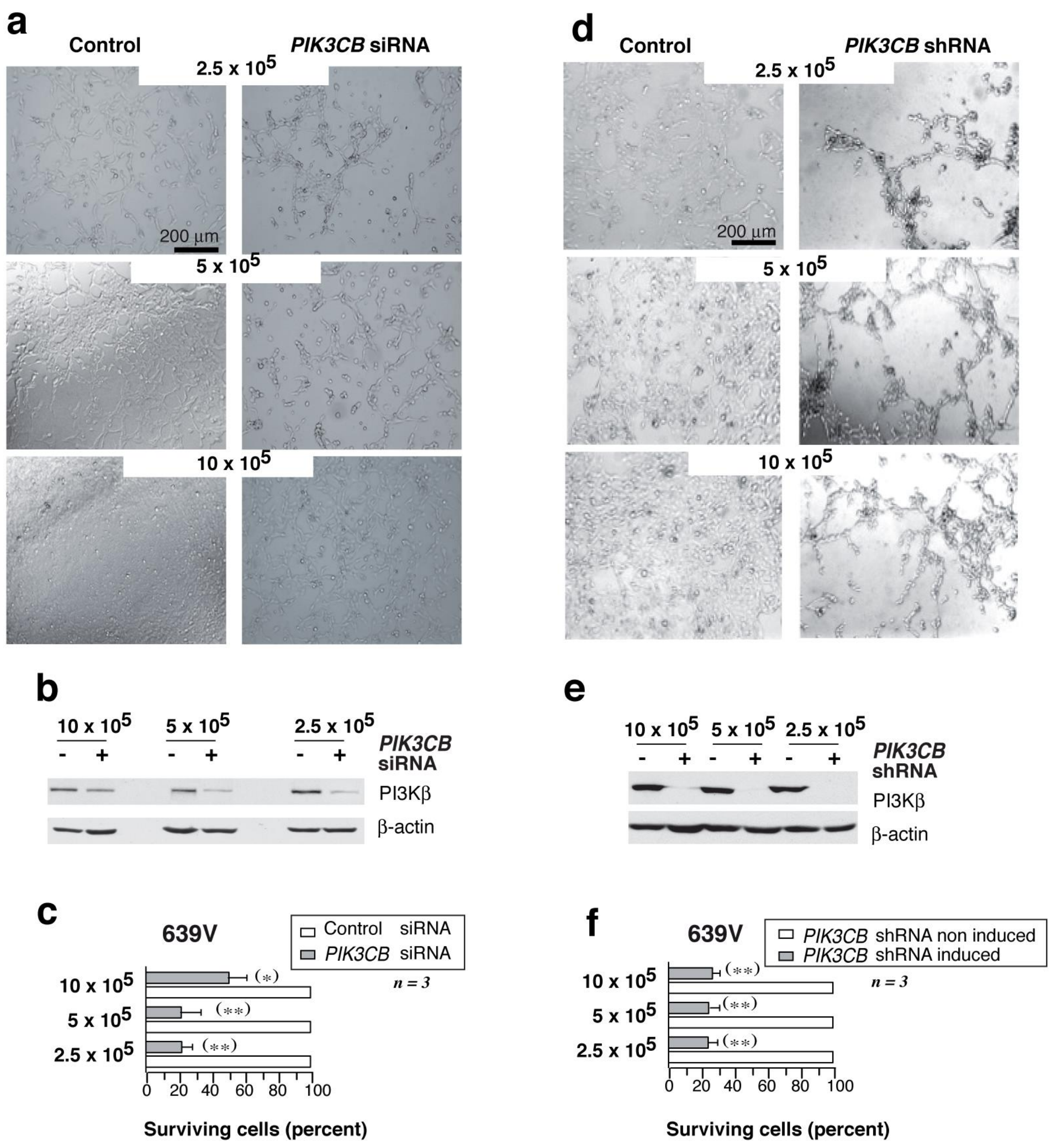

Suppl. Figure S2. Optimization of *PIK3CB* depletion in 639V cells. (a-c) Different numbers of 639V cells (indicated) were plated on p100 dishes, transfected on days 1 and 3 with 0.2 nmol control or si*PIK3CB*, and analyzed on day 5. Images show representative fields (a) and WB analysis of tumor extracts (b). The graph (c) shows the percentage of surviving cells at experiment termination in siPIK3CB- compared to control siRNA (scrambled)-transfected cells (100%). (**d-f**) 639V clones (different cell numbers, indicated) expressing doxycycline-inducible *PIK3CB* shRNA were plated on p100 dishes, allowed to attach, and treated with doxycycline (5 µg/ml; 72 h). Images show representative fields (d) and WB analysis of tumors extracts (e). Graph (f) shows the percentage of surviving cells as in (c). ** $P < 0.01$, * $P < 0.05$; Student's t-test.

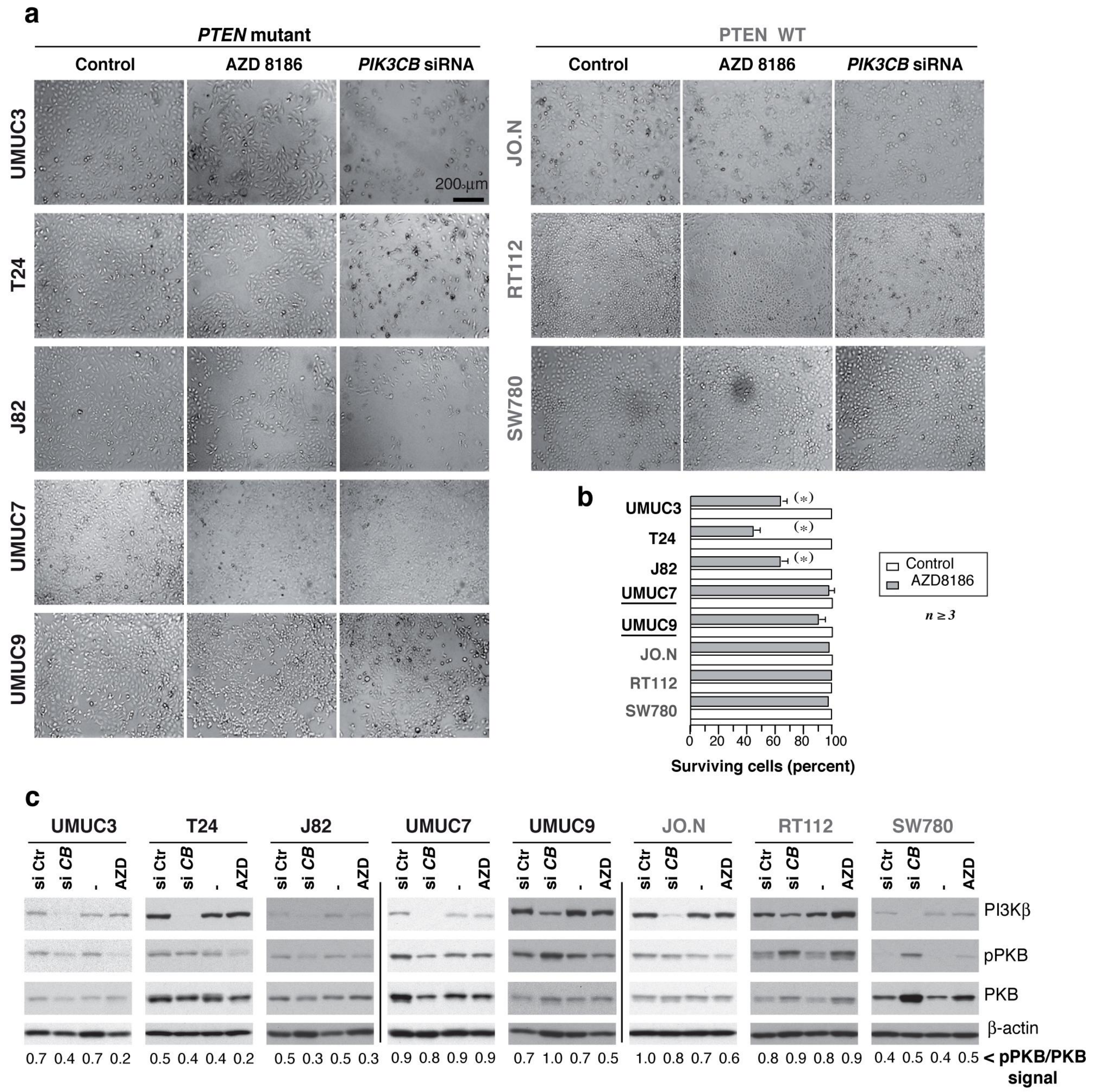

Suppl. Figure S3. PI3Kβ depletion has a greater effect on UBC cell survival than PI3Kβ inhibition. **(a-c)** Bladder carcinoma cell lines (indicated) were treated with 250 nM AZD8186 (72 h) or transfected with siPIK3CB, and analyzed as in Figure 3a. Images show representative fields (a) and percentage of surviving cells at experiment termination compared to control (vehicle) cells (100%) (b). Extracts of the different cell treatments were examined in WB with various antibodies (c). pPKB signal was normalized to that of PKB and is indicated below the blots. * P <0.05; Student's t-test.

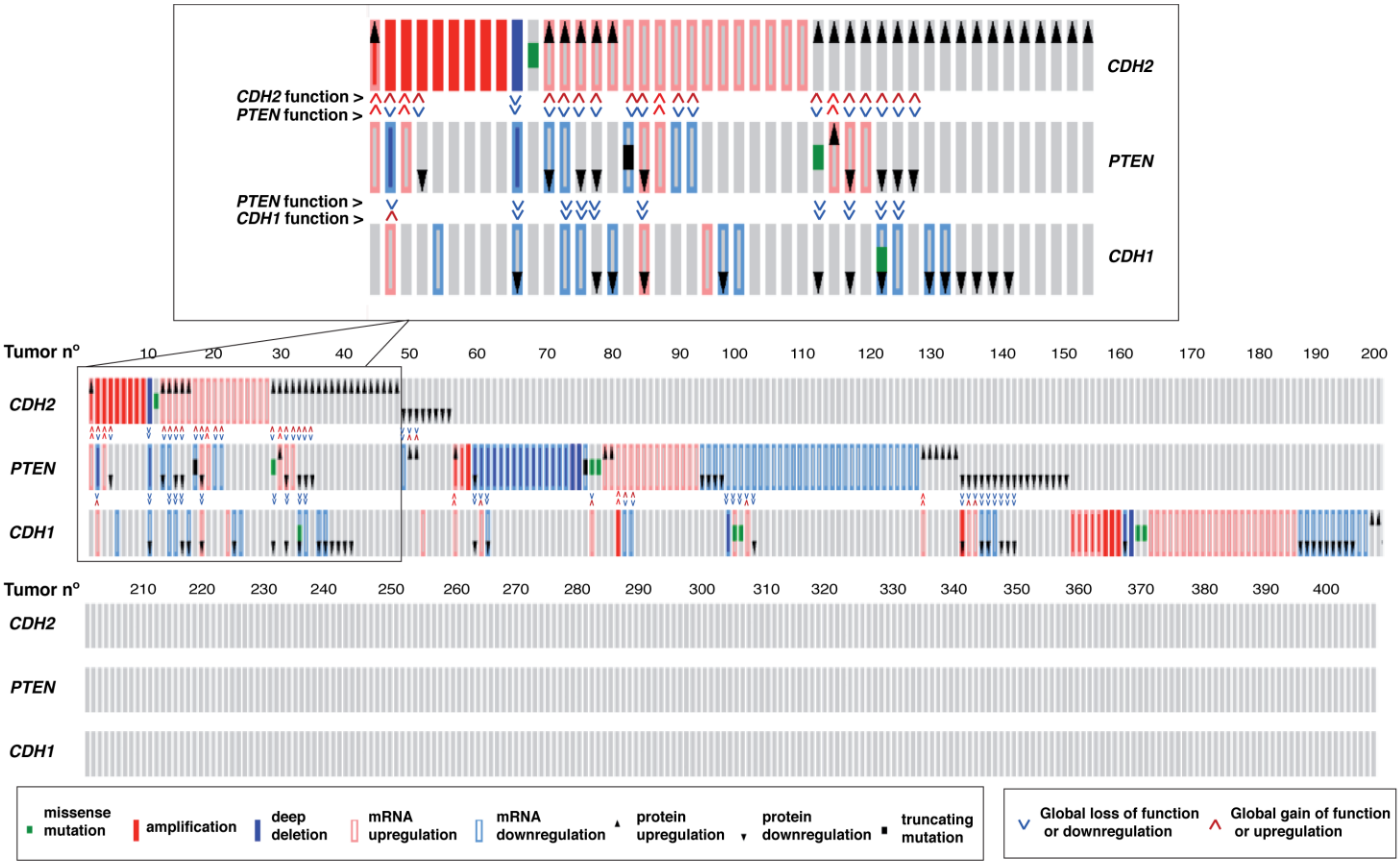

Suppl. Figure S4. ***PTEN*** **loss of function has a tendency to coincide with** ***CDH2*** **upregulation and reduced** ***CDH1*** **expression in UBC clinical samples.** Mutation and expression changes in *PTEN, CHD1* and *CHD2* genes in UBC samples from the Cancer Genome Atlas (TCGA) cancer collection (cancer genome.nih.gov), studied with cBioportal software (cbioportal.org/index.do). Each grey bar represents a tumor sample, we used the cBioportal legends within grey bars (indicated). Red and blue arrowheads outside grey bars indicate global gain of function/upregulation (red) or loss of function/down-regulation (blue) in samples with simultaneous alterations in *PTEN* and *CHD2* or in *PTEN* and *CHD1.*

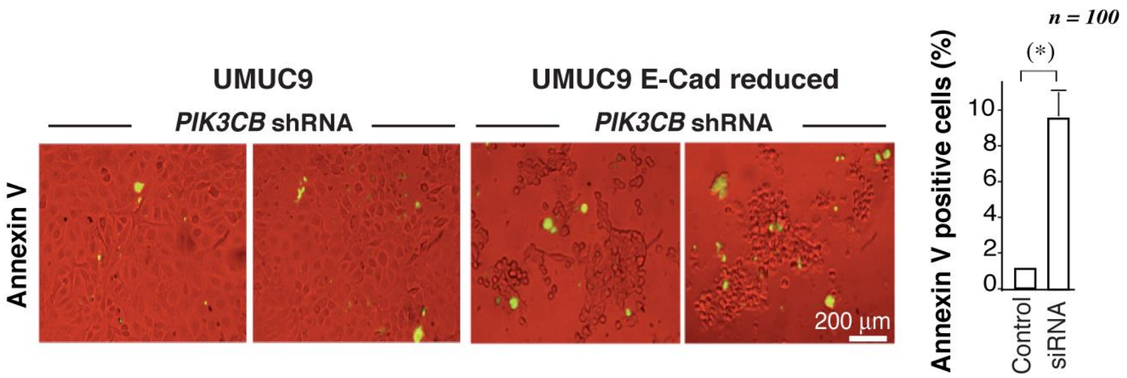

Suppl. Figure S5. E-cad-depleted UMUC-9 cells are sensitized to *PIK3CB* depletion. Control or E-cad-depleted UMUC-9 cells treated with *PIK3CB* shRNA (96 h) were annexin-V -stained; representative images and percentage of positive cells. * P <0.05; Student's t-test.

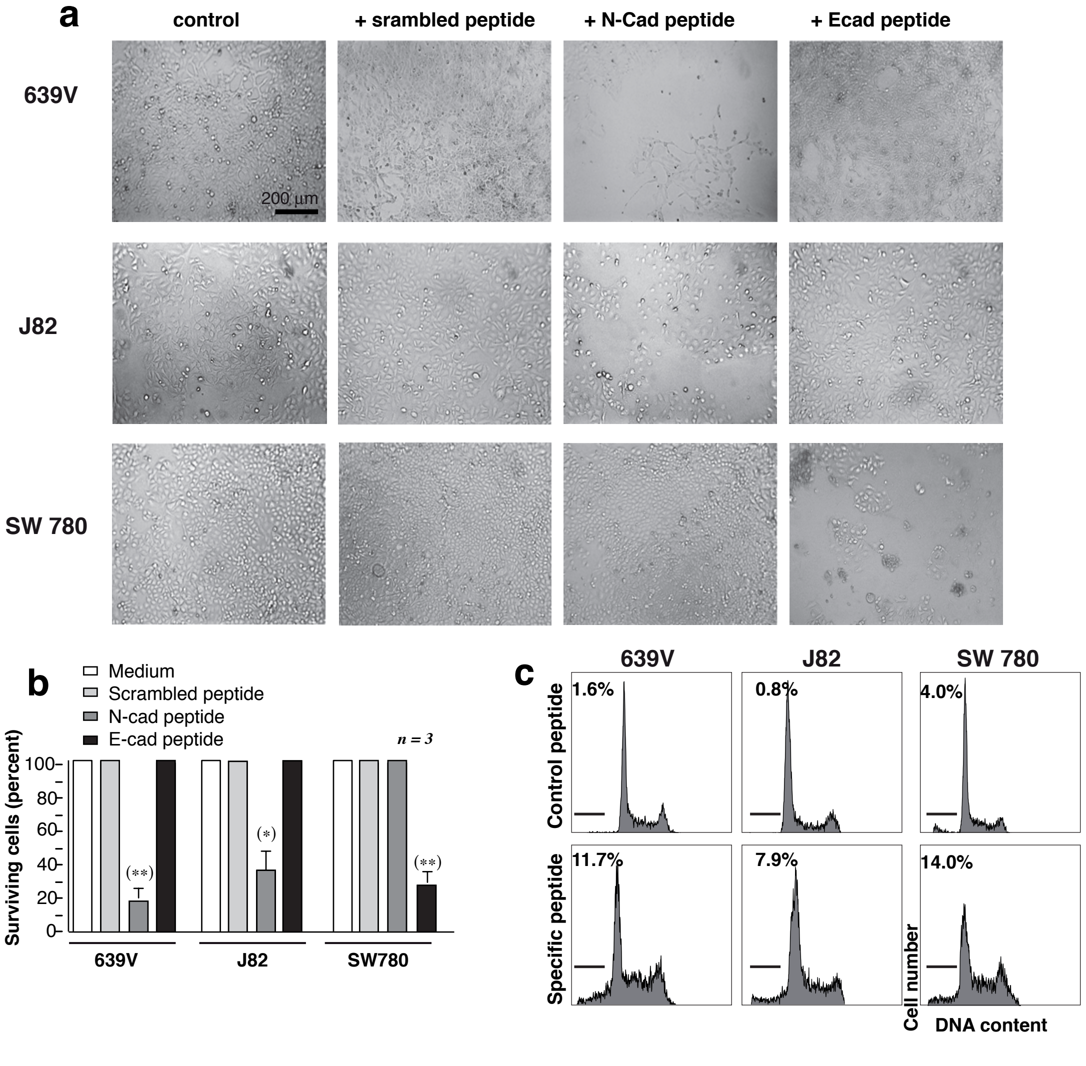

Suppl. Figure S6. Disruption of cell-cell adhesions impairs UBC cell survival. (a-c) Formation of cadherin-based junctions was inhibited using two synthetic peptides that interfere with E-cad-E-cad or N-cad-N-cad interactions, or a scrambled peptide as control. UBC cell lines (639V, J82, SW780) were cultured with the indicated peptides (400 µg/ml; 72 or 96 h). (a) Representative images at experiment termination (96 h). (b) Percentage of surviving cells relative to controls. (c) Cell cycles of cells untreated or treated with N-cad or E-cad peptides (72 h). The percentage of cells with sub-G1 DNA content is indicated. E-cad peptide selectively inhibited E-cad-expressing cells, whereas N cad peptide selectively blocked N-cad-expressing cell survival. ** P <0.01, *P <0.05; Student’s t-test.
